# Unraveling the regulatory cell death pathways in gastric cancer: a multi-omics study

**DOI:** 10.3389/fphar.2024.1447970

**Published:** 2024-09-09

**Authors:** Jiazheng Sun, Lixiang Rao, Sirui Zhou, Yulan Zeng, Yalu Sun

**Affiliations:** ^1^ Liyuan Hospital, Tongji Medical College, Huazhong University of Science and Technology, Wuhan, China; ^2^ Affiliated Hospital of Jining Medical University, Jining, China

**Keywords:** gastric cancer, regulatory cell death, immune microenvironment, prognostic signature, immunotherapy

## Abstract

Gastric cancer (GC) is a prevalent form of cancer worldwide and has a high death rate, with less than 40% of patients surviving for 5 years. GC demonstrates a vital characteristic of evading regulatory cell death (RCD). However, the extent to which RCD patterns are clinically significant in GC has not been well investigated. The study created a regulatory cell death index (RCDI) signature by employing 101 machine-learning algorithms. These algorithms were based on the expression files of 1292 GC patients from 6 multicenter cohorts. RCDI is a reliable and robust determinant of the likelihood of surviving in general. Furthermore, the precision of RCDI surpasses that of the 20 signatures that have been previously disclosed. The presence of RCDI signature is closely linked to immunological characteristics, such as the infiltration of immune cells, the presence of immunotherapy markers, and the activation of immune-related functions. This suggests that there is a higher level of immune activity in cases with RCDI signature. Collectively, the use of RCDI has the potential to be a strong and encouraging method for enhancing the clinical results of individual individuals with GC.

## 1 Introduction

Gastric cancer (GC) is a widespread malignancy with a rapidly increasing incidence each year (Thrift and El-Serag, 2020). GC is the fifth most common cancer worldwide in terms of new cases and the fourth leading cause of death globally, according to the 2020 Global Cancer Epidemiology statistics. In China, GC is the third most prevalent type of cancer, with approximately 480,000 new cases. It is responsible for 12.4% of all cancer-related fatalities in the country (Sung et al., 2021). This is directly associated with the high prevalence of GC patients in China and the significant variability in tumor biology and clinical characteristics. Forecasting the outcome of GC poses a significant obstacle in the current clinical approach to GC treatment.

Escape from cell death is a crucial trait exhibited by tumors (Tan et al., 2021). Regulatory cell death (RCD) is a form of cell death that happens when signal transduction modules are triggered to maintain the stability of the internal environment (Tang et al., 2019). Studies have found that numerous pathways involved in regulatory cell death signaling play a role in the development and advancement of GC. Studying these pathways will help to progress the development of diagnostic and treatment methods for GC.

Rayes et al. (2019) verified that the process of neutrophil-induced NETosis contributed to facilitating the spread of GC cells. Hao et al. (2017) reported that suppressing the expression of cysteine dioxygenase 1 in GC cells can replenish the level of glutathione (GSH), enhance the activity of glutathione peroxidase 4 (GPX4), hinder the generation of reactive oxygen species (ROS), and impede iron-induced cell death. Moreover, elevated concentrations of arachidonic acid and adrenic acid in GC facilitate the production of polyunsaturated fatty acids (PUFA), which trigger lipid peroxidation and accelerate iron-mediated cell death (Lee et al., 2020). Guo et al. (2019) found that celastrol, which has anticancer properties, elevated the levels of phosphorylation of RIP1 and RIP3. This resulted in the necrotic death of GC cells. The studies collectively indicate that RCD plays a crucial role in both the formation and advancement of GC.

**FIGURE 1:**
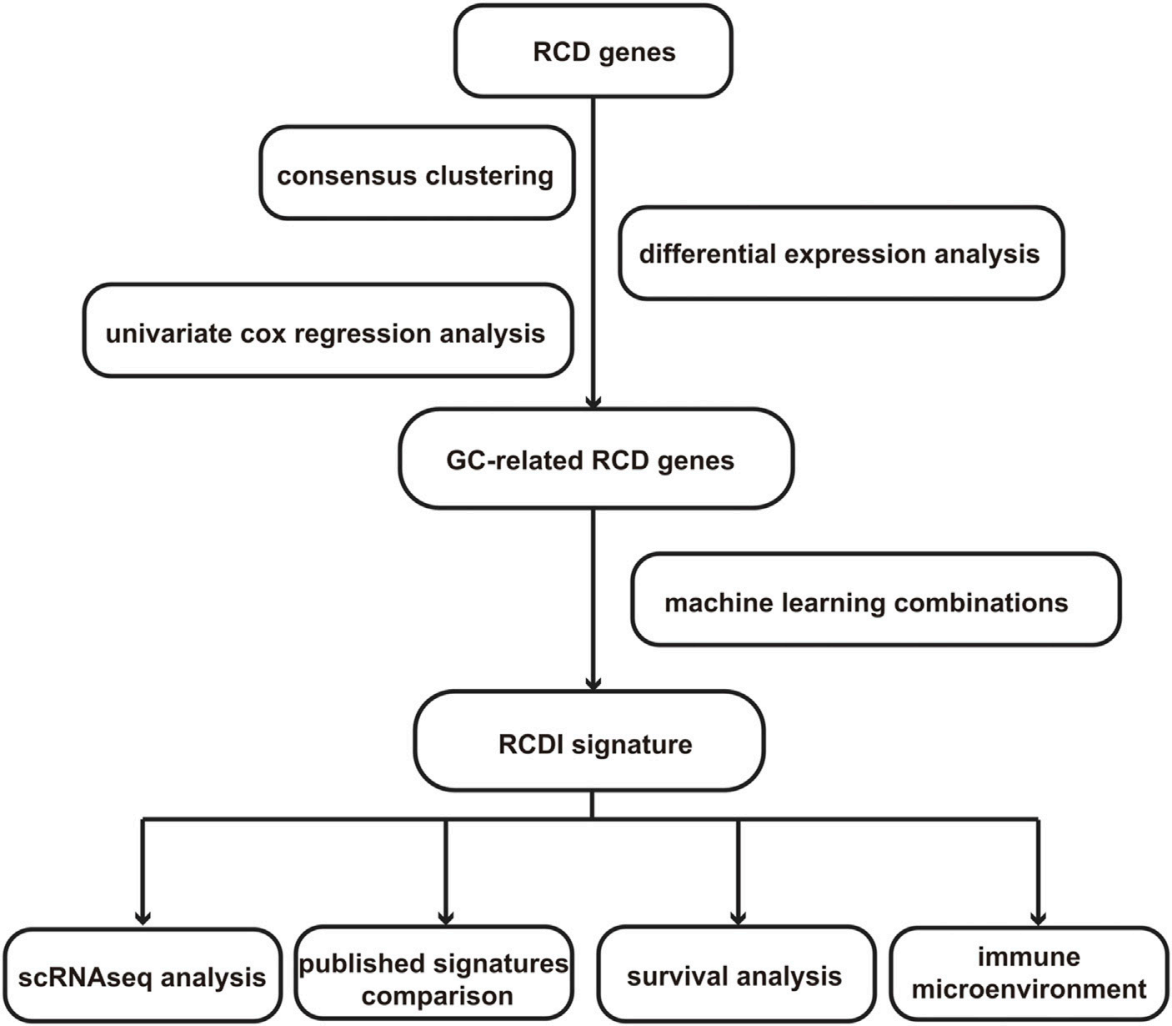
The study’s flowchart diagram.

However, the majority of the previously mentioned research solely examines the influence of a specific RCD mode on GC. Currently, there is a lack of extensive understanding of the interactions among RCD patterns in GC. Additionally, there is a limited quantity of particular studies on the functional aspects of these processes in GC. To tackle these regions of low comprehension, we have developed a new metric called the regulatory cell death index (RCDI). This metric is specifically formulated to predict the efficacy and prognosis of treatment strategies for GC. We identified heterogeneity in patients with GC and assessed their clinical prospects based on the RCDI signature, which provides valuable guidance for selecting the most effective treatment. The specific process of the study is shown in Figure 1.

## 2 Materials and methods

### 2.1 Data acquisition and processing

The gene expression data, consisting of 375 GC samples and 32 samples of para-cancer tissue, along with the clinical data of GC patients, were obtained from the TCGA database (https://www.tcga.org) (Cancer Genome Atlas Research Network, 2014). The bulk-seq datasets including GSE84437 (Yoon et al., 2020), GSE13861 (Cho et al., 2011), GSE15459 (Tao et al., 2011), GSE26253 (Oh et al., 2018), GSE26901 (Oh et al., 2018), and GSE57303 dataset (Qian et al., 2014) as well as scRNA-seq GSE183904 dataset (Kumar et al., 2022) were obtained from the Gene Expression Omnibus (GEO) database (http://www.ncbi.nlm.nih.gov/geo/).

Ultimately, the study encompassed 6 unique cohorts of patients, totaling 1,292 individuals diagnosed with GC, in order to establish the prognostic signature. The criteria for inclusion were as follows: 1) The individuals were diagnosed with GC. 2) The patients possess comprehensive survival statistics (Refer to Supplementary Table S1 for comprehensive clinical parameters). The GSE84437 cohort is designated as the training cohort. The validation cohort consists of the following datasets: GSE13861, GSE15459, GSE26253, GSE26901, and GSE57303 cohort.

PRJEB25780 dataset including the immunotherapy information and RNAseq data of 78 GC patients was obtained from a published study (Kim et al., 2018) (Refer to Supplementary Table S2 for details).

### 2.2 Functional enrichment analysis

Gene Ontology (GO) enrichment analysis is a widely used method for conducting large-scale investigations on the functional enrichment of biological processes (BPs), molecular functions (MFs), and cell components (CCs). KEGG enrichment analysis, a widely used bioinformatics technique, is employed to examine gene pathways and functional enrichment in a certain gene set. The above enrichment analysis was performed based on the “clusterProfiler” R package.

### 2.3 Consensus clustering

The “ConsensusClusterPlus” R package (Wilkerson and Hayes, 2010) was employed to find the cluster of RCD-related genes based on the expression data of RCD-related genes in the GSE84437 cohort and all samples. Subsequently, the optimal number of clusters was determined using the consensus score matrix, CDF curve, PAC score, and Nbclust.

### 2.4 Integration of machine learning algorithms

To enhance the precision and consistency of the RCDI signature, the study incorporated ten machine-learning algorithms into our analysis. These algorithms encompass Lasso (Friedman et al., 2010), CoxBoost (Binder et al., 2009), random survival forest (RSF) (Rigatti, 2017), elastic network (Enet) (Friedman et al., 2010), Ridge (Friedman et al., 2010), Stepwise Cox (Núñez et al., 2011), partial least squares regression for Cox (plsRcox) (Lê Cao et al., 2011), supervised principal components (SuperPC) (Bair and Tibshirani, 2004), generalized boosted regression modeling (GBM) (Guo and Chang, 2022), and survival support vector machine (survival-SVM) (Van Belle et al., 2011). Among these algorithms, Lasso, stepwise Cox, CoxBoost, and RSF have shown feature selection capabilities. Therefore, we integrated these algorithms to produce a consensus model. A total of 101 algorithm combinations were performed to construct prediction models using the 10-fold cross-validation technique.

### 2.5 Collection of biomarkers in cancer immunotherapy

The relationship between the RCDI signature and immune cell infiltration in tumor immune microenvironment (TIME) was investigated based on the TIMER algorithm (Li et al., 2017), CIBERSORT algorithm (Newman et al., 2019), quantiseq algorithm (Finotello and Trajanoski, 2018), MCPcounter algorithm (Becht et al., 2016), and EPIC algorithm (Racle et al., 2017).

Furthermore, seven published immunotherapeutic biomarkers were enrolled. The “easier” package (Lapuente-Santana et al., 2021) was used to calculate Cytotoxic activity (CYT) (Rooney et al., 2015), IFNy signature (IFNy) (Ayers et al., 2017), Roh immune score (Roh_IS) (Roh et al., 2017), chemokine signature (chemokines) (Messina et al., 2012), Davoli immune signature (Davoli_IS) (Cabrita et al., 2020), extended immune signature (Ayers_expIS) (Ayers et al., 2017). TIDE scores were retrieved from the TIDE database (http://tide.dfci.harvard.edu/).

### 2.6 Drug sensitivity analysis

The “pRRophetic” R package was applied to predict the therapeutic response of GC patients to common drugs, and the value of the RCDI signature in guiding the selection of drugs for GC patients was assessed based on the IC50 values in different RCDI score groupings.

### 2.7 Single-cell analysis

The scRNA-seq data of GSE183904 were analyzed using the R package “Seurat” (4.0.3). Cells with <300 genes, or >20% mitochondrial genes were excluded. Subsequently, this study performed Principal Component Analysis (PCA) using the first 1,500 highly variable genes. The first 15 principal components were then chosen to construct the t-Distributed Stochastic Neighbor Embedding (t-SNE) plot. Clustree graphically represents the Seurat resolution concerning cluster clusters, providing guidance on the number of cell clusters and selecting the most suitable level of resolution. The “FindAllMarkers” function retrieves the hallmark genes associated with each cell cluster.

Cell clusters were annotated using reference data from the Human Cell Atlas and were subsequently refined based on specific cell biomarkers including Epithelial cells (EPCAM, KRT18, and MUC1) Endothelial cells (VWF) Fibroblasts (LUM) Plasma cells (MZB1) NK/T cells (CD2 and NKG7) B cells (CD79A and MS4A1) Macro/Mono (CD14 and CD68) and Mast cells (MS4A2).

The “infercnv” R package was utilized to analyze the copy number variation (CNV) of epithelial cells. Predictions were made about the existence of malignant epithelial cells and normal epithelial cells based on the CNV score. The “irGSEA” R package was employed to conduct gene set enrichment analysis (GSEA) on the scRNAseq dataset using the “AUCell”, “UCell”, “singscore”, and “ssgsea” algorithms.

### 2.8 The protein expressions of prognostic genes

The protein expression of the prognostic hub genes between GC and normal tissues was validated using immunohistochemistry (IHC) provided by the Human Protein Atlas database (HPA, https://www.proteinatlas.org/).

### 2.9 Statistical analysis

The R package “limma” was employed to extract differentially expressed genes (DEGs). Statistical differences between groups were determined by Student’s t-test for normally distributed variables, and for non-normally distributed variables, statistical differences between groups were determined by the Wilcoxon test. The statistical studies were conducted using the R project (version 4.3.3).

## 3 Results

### 3.1 Variant landscape of RCD-related genes in GC patients

**FIGURE 2:**
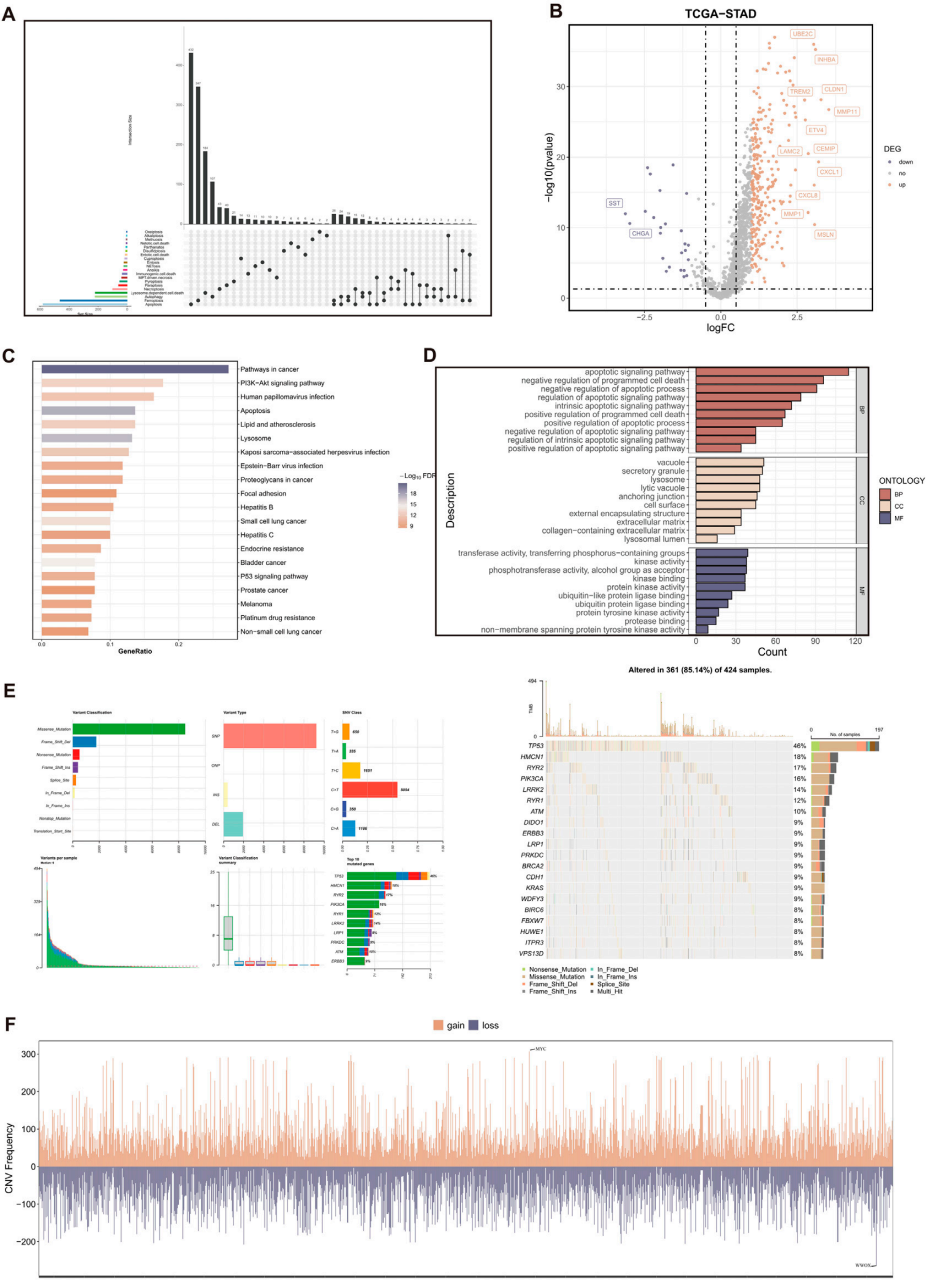
Variant landscape of RCD-related genes in GC patients. **(A)** The upset plot displaying diverse RCD patterns and key regulatory genes. **(B)** Volcano plot of the RCD-related DEGs. Points with labels are obvious DEGs which adjusted. *p*-value < 0.05 and |log2FC| > 1. **(C)** GO enrichment analyses based on the DEGs. **(D)** KEGG enrichment analyses based on the DEGs. **(E)** An oncoplot of RCD-related genes in the TCGA-STAD cohort. **(F)** CNV values of RCD-related genes in the TCGA-STAD cohort.

In this study, we collected a total of 20 RCD patterns and 2013 key regulatory genes (Refer to Supplementary Table S3 for details) from the existing published articles (Qin et al., 2023; Liu et al., 2023a; Liu et al., 2023b), MSigDB Database (http://software.broadinstitute.org/gsea/msigdb/index.jsp), KEGG database (https://www.genome.jp/kegg), and Gene cards database (https://www.genecards.org/). We removed 449 duplicate gene symbols, resulting in 1564 RCD-related genes for subsequent analysis. (Figure 2A).

A total of 285 genes with significant differential expression (adjusted *p*-value < 0.05 and |log2FC| > 1) were identified in the TCGA-STAD cohort (Figure 2B). Furthermore, the DEGs are associated with many cell death pathways and signal transduction pathways linked to cancer, as demonstrated by the KEGG and GO enrichment studies (Figures 2C,D). The TCGA-STAD cohort was used to assess the variation in RCD-related genes.

The findings indicated that approximately 85.14% (361 out of 424) of individuals with GC exhibited genetic alterations. Figure 2E displayed the top 20 mutations in RCD-related genes, with TP53 exhibiting the highest mutation frequency at 46%. The examination of CNV status revealed frequent alterations in RCD-related genes. Analysis revealed that WWOX had the highest degree of CNV deletion, whereas MYC displayed the most pronounced CNV amplification (Figure 2F).

### 3.2 Identification of RCD-related genes associated with GC

**FIGURE 3:**
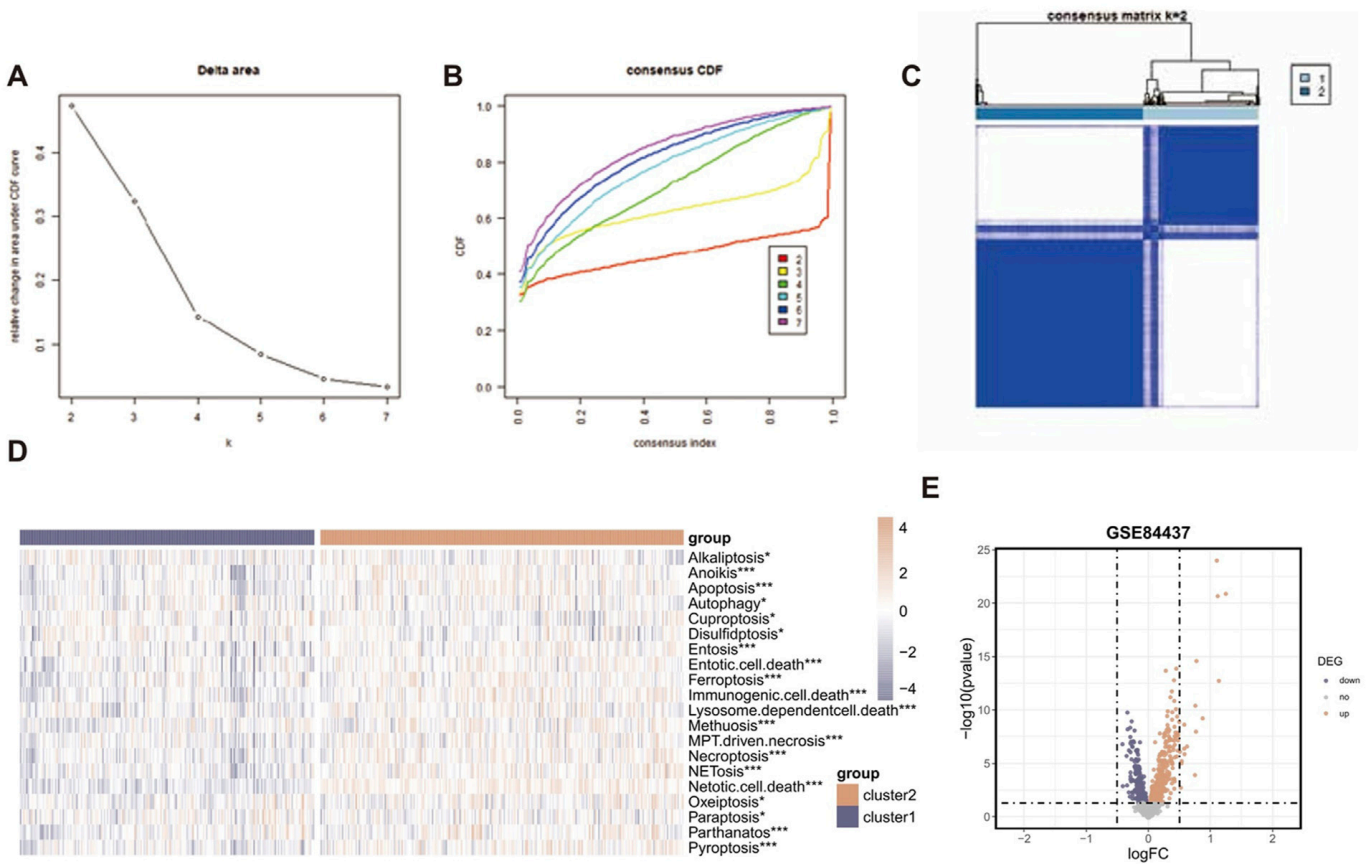
Identification of RCD-related genes associated with GC. **(A)** Consensus clustering model with cumulative distribution function (CDF) for k = 2–7 (k means cluster count). **(B)** Relative change in the area under the CDF curve for k = 2–7. **(C)** The consensus score matrix of all samples when k = 2. A higher consensus score between two samples indicates they are more likely to be grouped into the same cluster in different iterations. **(D)** The heatmap displaying the heterogeneity in RCD-related pathway activity between two clusters based on the ssGSEA algorithm. **(E)** The volcano plot of the RCD-related DEGs. Points with labels are obvious DEGs with adjusted. *p*-value < 0.05.

The “ConsensusClusterPlus” R package was employed to conduct consensus cluster analysis on a set of 1,564 RCD-related genes. This study utilized a uniform clustering approach to divide the GC data into k clusters, with k ranging from 2 to 7. The cumulative distribution function (CDF) curve of the consensus score matrix and the proportion of ambiguous clustering (PAC) statistics for the fuzzy clustering ratio suggest that the optimal number of clusters is attained when k = 2 (Figures 3A,B). Two distinct subcategories of RCD, namely, Cluster A and Cluster B, were identified, (Figure 3C). Figure 3D illustrates the heterogeneity in RCD-related pathway activity between two clusters based on the ssGSEA algorithm. A total of 859 RCD-related genes that showed differential expression were identified when comparing the two RCD subtypes (*p*-value < 0.05) (Figure 3E).

### 3.3 Construction and validation of the RCDI signature

**FIGURE 4:**
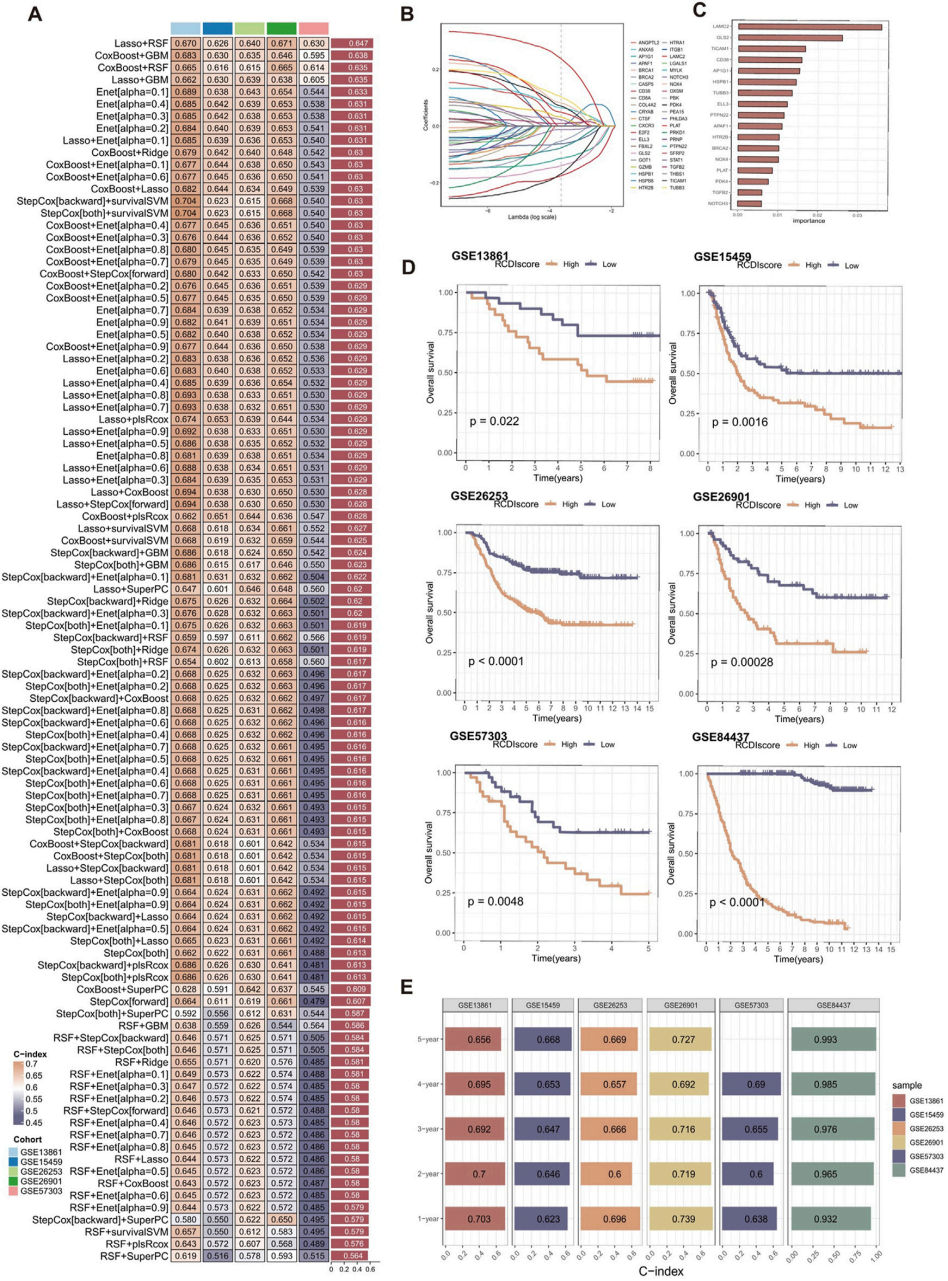
Construction and validation of the RCDI signature. **(A)** A total of 101 combinations of machine learning algorithms for the RCDI signatures via a 10-fold cross-validation framework based on the GSE84437 cohort. The C-index of each model was calculated across validation datasets, including GSE13861, GSE15459, GSE26253, GSE26901, and GSE57303 cohort. **(B)** The coefficients of the most useful prognostic genes based on the lasso algorithm in the GSE84437 cohort. **(C)** The importance of the 17 most valuable genes based on the RSF algorithm in the GSE84437 cohort. **(D)** Kaplan-Meier survival curve of OS between patients in high-RCDI score group and low-RCDI score group in GSE13861, GSE15459, GSE26253, GSE26901, GSE57303, and GSE84437 cohort. **(E)** ROC analysis of RCDI in GSE13861, GSE15459, GSE26253, GSE26901, GSE57303, and GSE84437 cohort.

The best-performing predictive signature was determined as the signature with the greatest mean C-index in five external validation cohorts, due to overfitting in the training cohort (Figure 4A). The findings indicated that the Lasso + RSF algorithm combination demonstrated the highest average C-index (0.647), making it the optimal combination of algorithms for developing the RCDI signature.

Lasso algorithm was used to screen out the most valuable genes (Figure 4B). RSF algorithm was further used to filtrate the most reliable model (Figure 4C). Log-rank score test for splitting survival trees was conducted. First, the x-variable x was assumed to be ordered as $x_{1}\leq x_{2}...\leq x_{n}$ . Then, the “ranks” for each survival time $T_{j}\left(j\;\epsilon \;\left[1,...,n\right]\right)$ were computed. The obtained equation is as follows:

$$a_{j}=\delta_{j}-\sum_{k=1}^{\Gamma_{j}}\frac{\delta_{k}}{n-\Gamma_{k}+1}$$

where $\Gamma_{k}=\#\left[t:T_{t}\leq T_{k}\right]$ and $\Gamma_{j}$ represents the index of the order for $T_{j}$ . The log-rank score test came as follows:

$$\text{RCDI signature}=S\left(x,c\right)=\frac{\sum_{\text{xk}\leq c}\left(a_{j}-n_{l}\bar{a}\right)}{\sqrt{n_{l}\left(1-\frac{n_{l}}{n}\right)}S_{a}^{2}}$$

where $\bar{a}$ and $S_{a}^{2}$ represent the sample mean and sample variance of $\left[a_{j}:j=1,...,n\right]$ , respectively. The measure of node separation is determined using log-rank score splitting by $\left|S\left(x,c\right)\right|$ . The best split is reached by maximizing this value over x and c.

Afterwards, the RCDI score for each sample was calculated. Kaplan-Meier analysis and assessment of prognostic performance were conducted. Demonstrating a substantial difference in survival time between the low-RCDI score and high-RCDI score groups in all six cohorts (Figure 4D). The accuracy and reliability of the RCDI signature predicting 1-, 2-, 3-, 4- and 5-year survival of GC patients was supported by empirical evidence that the area under the curve (AUC) values exceeded 0.65 in multiple distinct cohorts (Figure 4E).

### 3.4 The relationship between RCDI signature and TIME characteristics

**FIGURE 5:**
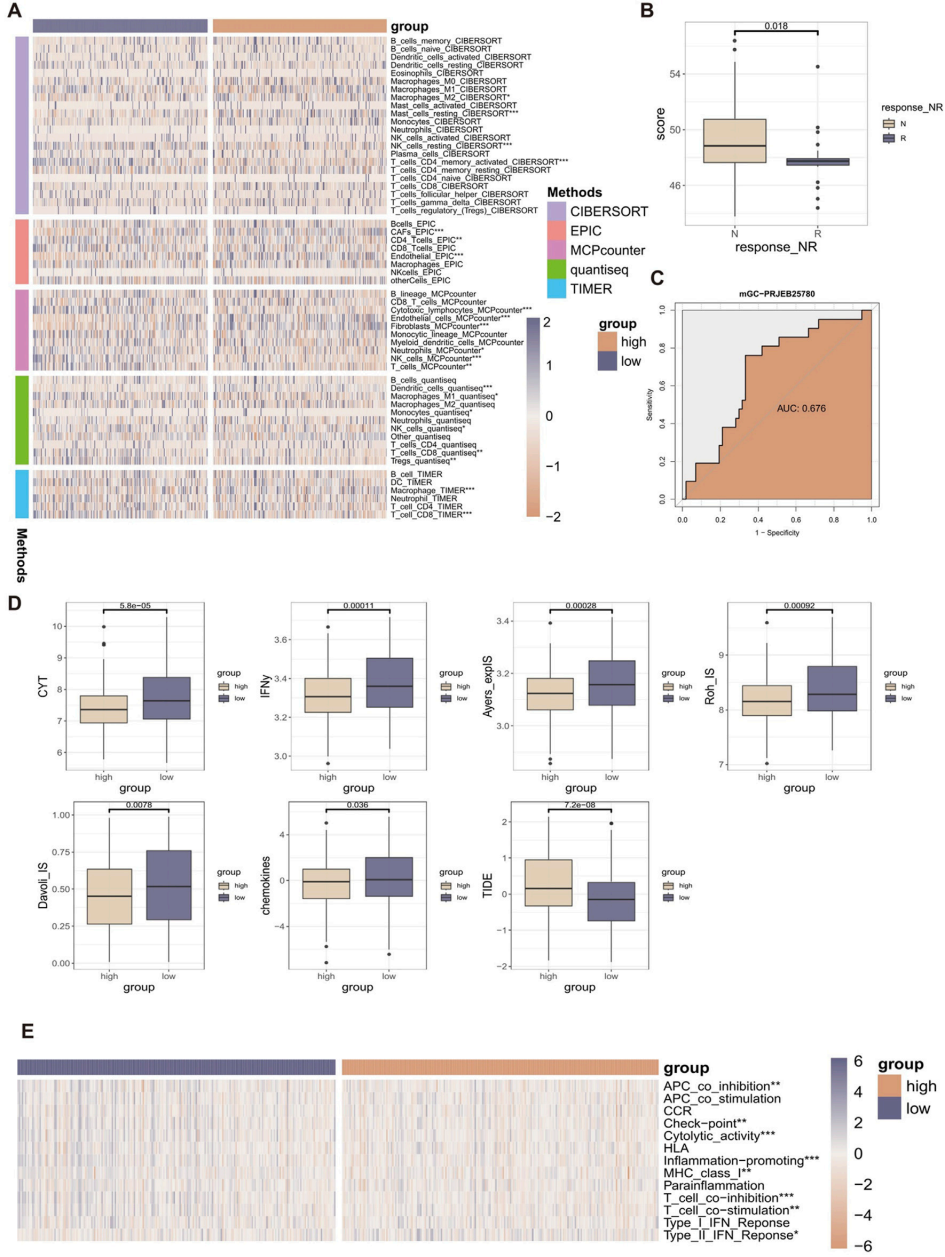
The relationship between RCDI signature and TIME characteristics. **(A)** Heatmap displaying the correlation between the RCDI signature and immune infiltrating cells. **(B)** Box plot displaying the correlation between the RCDI signature and immunotherapy response in the immunotherapy cohort. **(C)** ROC curves of the RCDI signature to predict the benefits of immunotherapy in the immunotherapy cohort. **(D)** Box plot displaying the correlation between the RCDI signature and immune modulators. **(E)** Heatmap displaying the correlation between the RCDI signature and 13 immune-related processes.

To assess the role of RCDI signature in GC TIME, we assessed the relationship between RCDI signature and immune infiltrating cells (Figure 5A). Based on the TIMER algorithm, CIBERSORT algorithm, quantiseq algorithm, MCPcounter, and EPIC algorithm, the RCDI signature was correlated with the majority of tumor immune infiltrating cells. The immune heterogeneity between the high-RCDI score group and the low-RCDI score group was demonstrated.

We also evaluated the predictive value of RCDI signature in immunotherapy using immunotherapy-related datasets. We found that in the PRJEB25780 cohort, The RCDI score of responders was significantly lower than that of non-responders (Figure 5B). The receiver operating characteristic (ROC) analysis showed that the RCDI signature exhibited a superior ability to predict the efficacy of immunotherapy-based treatment (Figure 5C).

In addition, the study also evaluated the relationship between RCDI signature and known immune modulators (CYT, IFNy, Davoli_IS, Roh_IS, Ayers_expIS, chemokines, and TIDE) (Figure 5D). The values of most of the immune modulators (CYT, IFNy, Davoli_IS, Roh_IS, Ayers_expIS, and chemokines) were significantly higher in the low RCDI scores group. Meanwhile, the TIDE score was significantly lower in the low RCDI scores group. A low TIDE score indicates less likelihood of immune escape and a better response to immunotherapy. Based on the ssGSEA algorithm, the RCDI signature was significantly correlated with most immune-related processes (Figure 5E).

These findings indicated that GC patients with lower RCDI scores may experience more favorable outcomes from immunotherapy treatment.

### 3.5 Assessment and clinical application of the RCDI signature

**FIGURE 6:**
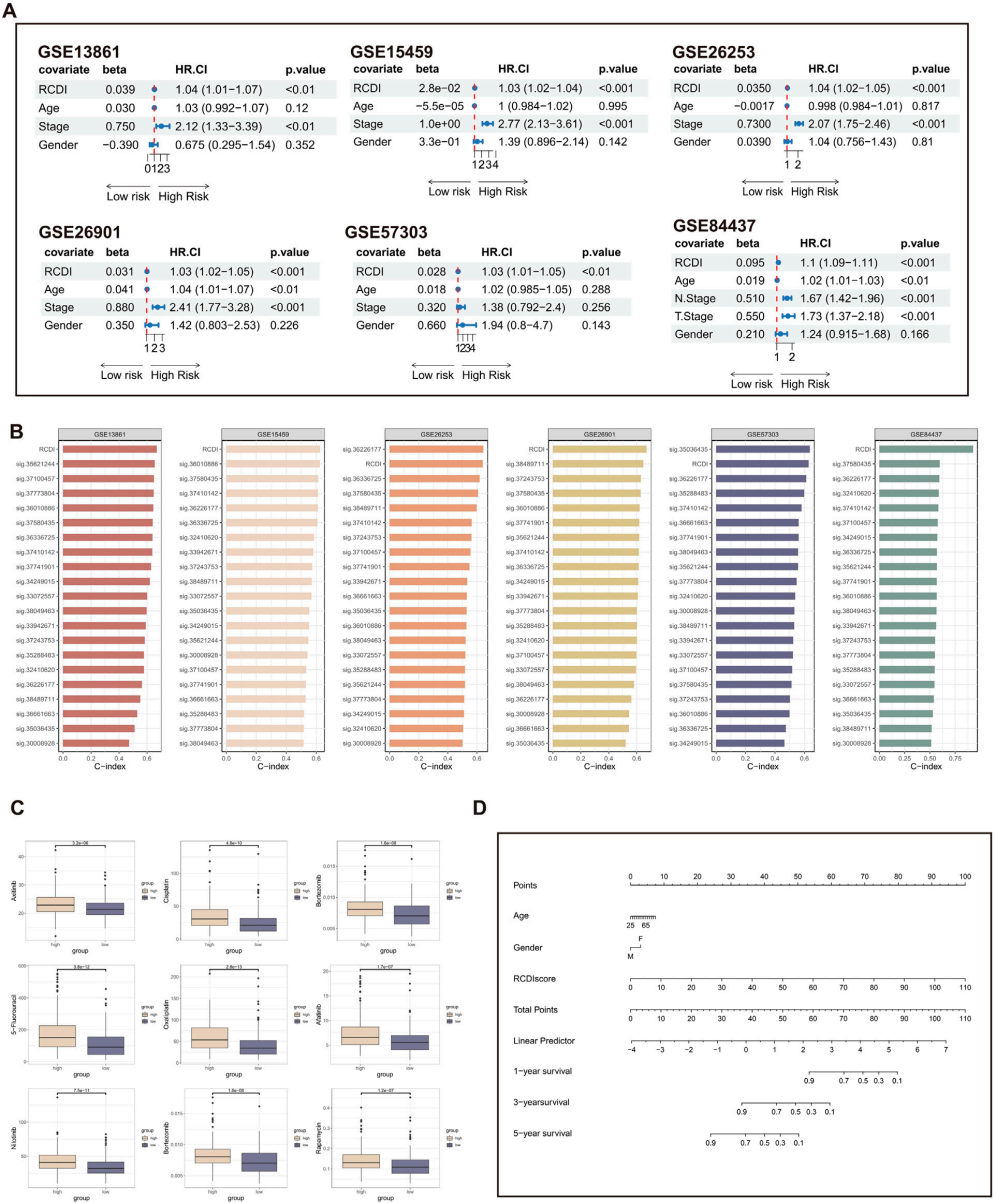
Assessment and clinical application of the RCDI signature. **(A)** Forest plot displaying associations between RCDI signature and other clinical features and the prognosis of GC based on univariate Cox regression analysis. **(B)** C-index comparison of RCDI signature and 20 previously published signatures. **(C)** Box plot displaying the correlation between the RCDI signature and chemotherapy drugs. **(D)** A nomogram was established to predict the prognostic of GC patients based on age, gender, and RCDI score.

Subsequently, we further performed univariate Cox regression for each characteristic of all cohorts. The results suggest that the RCDI signature may be utilized as an independent prognostic indication of unfavorable outcomes (Figure 6A).

Machine learning-based gene expression analysis can be utilized to forecast the onset of diseases, aiding in the early detection of diseases and facilitating research on novel treatments. Over the past few years, there has been a proliferation of disease prediction models related to GC. We searched literature about GC that focused on disease prediction models. After excluding papers that had an unclear formula for the prediction model and lacked gene expression data in both the training and validation groups, a total of 19 prediction models for GC-related diseases were collected (Supplementary Table S4). These traits encompass a range of metabolic and cell death mechanisms, such as cuproptosis, ferroptosis, and autophagy. The C-index of each signature in both the training cohort and the validation cohorts is computed and then compared with the C-index of the RCDI signature. The superiority of the RCDI signature is evident in comparison to the majority of signatures in each cohort (Figure 6B).

In addition, the drug sensitivity analysis revealed that GC patients with high RCDI scores saw a significant increase in their sensitivity to commonly used chemotherapeutic medicines for GC (Figure 6C). It is suggested that the RCDI signature has a potential guiding effect on the treatment of GC patients. Ultimately, to facilitate clinical application, a nomogram was created, integrating the factors of age, gender, and RCDI score (Figure 6D).

### 3.6 Dissection of tumor microenvironment based on RCD patterns

To identify the optimal resolution for unsupervised clustering, the effectiveness of 15 distinct resolution values was assessed by employing “clustree” R package (Supplementary Figure S1A). The authors selected a resolution of 0.5 for the initial distinction of cell kinds, as indicated by the pre-assigned notes. The tSNE plot identified 17 separate cell clusters, with each cluster being assigned a unique color (Supplementary Figure S1B). A total of 8 known cell types were identified based on specific cell marker genes (Supplementary Figures S1C,D).

**FIGURE 7:**
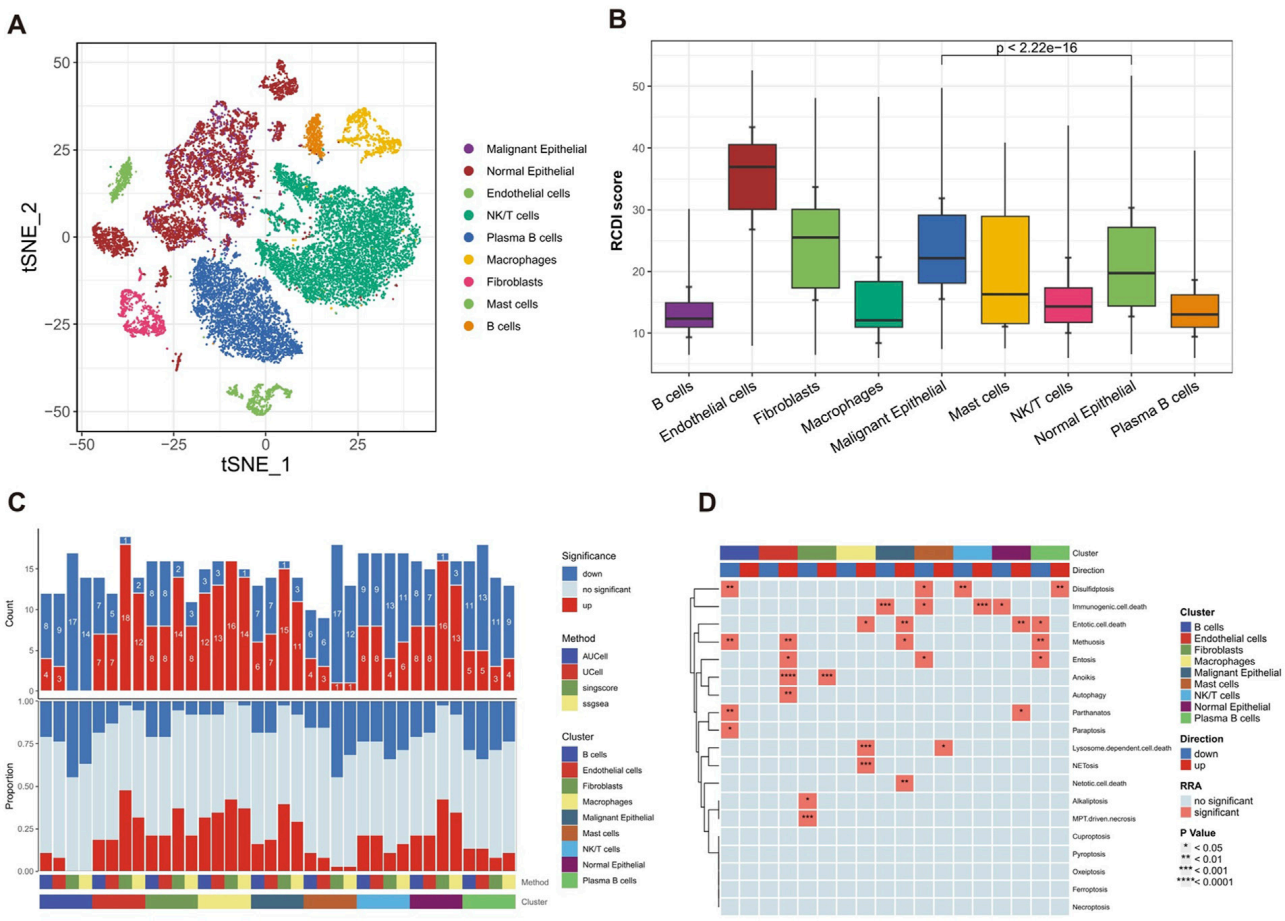
Heterogeneity of multiple RCD patterns in cell subsets in GC patients. **(A)** The t-SNE plot displaying the composition of cells in the microenvironment of GC. **(B)** The boxplot displaying the RCDI score in different cell types. **(C)** Histogram displaying the number of RCD patterns in different cell types. **(D)** The heatmap displaying the RCD patterns in different cell types.

Then, epithelial cells were isolated and subsequently reaggregated into 14 clusters using the “Seurat” and “clustree” R packages (Supplementary Figures S2A,B). In order to identify malignant epithelial cells in GC, the “infercnv” R package was employed to analyze the CNV in each epithelial cell on a wide scale and quantify it as the CNV score, with fibroblasts and endothelial cells as reference (Supplementary Figure S2C). The results showed that the CNV scores of clusters 0, 1, 3–6, 8–9, and 12–13 were significantly higher than the reference cells and there was no significant difference in CNV scores between clusters 2, 7, 10, and 11 with reference cells (Supplementary Figure S2D). Therefore, clusters 0, 1, 3–6, 8–9, and 12–13 are categorized as malignant epithelial cells, whereas clusters 2, 7, 10, and 11 are categorized as normal epithelial cells (Supplementary Figure S2E). To summarize, a total of 9 unique cell types were effectively recognized and recorded (Figure 7A).

Furthermore, we explored the detailed distribution of RCDI scores in GC patients using single-cell RNA transcriptome data. We consistently found that malignant cells had a higher RCDI score than other cells (Figure 7B). This further explains why the higher RCDI score group had a worse prognosis.

Ultimately, the study preliminarily described the differences in RCD-related pathway activity of various cell subtypes in the GC microenvironment (Figures 7C,D).

## 4 Discussion

GC is a highly aggressive tumor characterized by a high rate of invasion, recurrence, and a bleak prognosis. In order to enhance the survival rate, it is necessary to conduct early screening of the factors associated with poor prognosis and provide tailored individual treatment. However, to achieve this, there is a need for more sensitive and accurate biomarkers. The current categorization of GC primarily relies on the histological features of tumor cells. Furthermore, numerous studies have demonstrated that molecular markers can also uncover clinical significance and predictive worth. As our knowledge of GC biology grows, researchers are exploring emerging prognostic criteria and novel molecular markers to improve prognosis accuracy and tailor treatment strategies.

The study employed scRNAseq and bulk-RNAseq to characterize the pathway activity profiling of RCD patterns in GC patients. In addition, 101 distinct machine-learning algorithm combinations were utilized to develop a stable RCDI signature, which was derived by analyzing comprehensive bulk-RNAseq datasets. The stability and reliability of the RCDI signature are ensured by utilizing the advantages of each algorithm and adopting the set learning technique. By conducting validation on several datasets, the signature demonstrated exceptional performance in forecasting the prognosis of GC patients.

Furthermore, the majority of genes enrolled in the RCDI signature have been confirmed to be involved in the progression of GC. The research of Yanrong et al. (Cui et al. 2020a) study suggested that NOTCH3 is a prognostic factor correlating with immune tolerance in GC. Furthermore, as a crucial target of miR-491-5p/miR-875-5p, NOTCH3 has been shown to promote gastric carcinogenesis by upregulating PHLDB2 expression and activating the Akt pathway (Kang et al., 2021). Song and Zhou (2021) discovered that HOXA10 facilitates the process of epithelial-mesenchymal transition, which contributes to the spread of GC. This is achieved, in part, through the regulation of the TGFB2/Smad/METTL3 signal transduction pathway. Zhang et al. (2022a) verified that the increased expression of PDK4 promoted the proliferation, migration, and invasion capacity of GC cells. Besides, Buckley et al. (2022) discovered that individuals with recessive mutations in the BRCA2 gene have a higher likelihood of developing GC. This finding suggests that GC could be considered part of the whole spectrum of cancer risks associated with the BRCA1/2 genes. Tang et al. (Buckley et al. 2022) reported that NOX4-driven ROS formation regulates the proliferation and apoptosis of GC cells through the GLI1 pathway. Furthermore, Tu et al. (2023) demonstrated that HTR2B Regulates Lipid Metabolism to Inhibit Ferroptosis in GC. Wang et al. (2022) reported that APAF1-binding long noncoding RNA promotes tumor growth and multidrug resistance in GC by blocking apoptosome assembly. Kwon et al. (2011) reported the frequent upregulation of LAMC2 in GC due to promoter demethylation. Similarly, Ii et al. (2011) discussed the co-expression of Laminin [β3 and γ2] chains and the epigenetic inactivation of the Laminin [α3] chain in GC. Peng et al. (37016377) reported FYN/TOPK/HSPB1 axis facilitates the proliferation and metastasis of GC. The research of Xu et al. (2020) suggested the downregulation of GLS2 has been linked to its role as a tumor suppressor gene in GC. In conclusion, these studies suggest that programmed cell death plays a critical role in the development and progression of GC, and RCDI signature could serve as a biomarker for assessing GC. Moreover, multiple studies have demonstrated that measuring the levels of TUBB3 in the serum can assist in determining the appropriate chemotherapy drugs for patients with advanced gastric cancer (Yu et al., 2012; Luo et al., 2015; Huang et al., 2013; Di Bartolomeo et al., 2021). TICAM1, also known as TRIF, is a protein involved in the TRIF-IFN-I pathway, which plays a role in Helicobacter-induced gastric cancer (Bali et al., 2024). Additionally, TICAM1 has been identified as a promising target for immune therapy in gastric cancer (Cui et al., 2020b). Zhang et al. (2022b) demonstrated that CD19^+^ CD24^hi^ CD38^hi^ regulatory B cells were higher significantly in patients with gastric cancer than in the healthy group. Ci et al. (2024) reported that AP1G1 has been shown to promote gastric cancer stem cell (GCSC) characteristics, indicating its involvement in shaping the properties of these cancer cells. Additionally, AP1G1 has been implicated in the inhibition of gastric cancer growth and tumor development, suggesting its potential as a therapeutic target for gastric cancer treatment (Ma et al., 2021). Furthermore, the downregulation of AP1G1 expression has been associated with the suppression of apoptosis in gastric cancer cells, further emphasizing the significance of AP1G1 in gastric cancer progression (Ci et al., 2024). Moreover, microRNAs have been implicated in gastric cancer, with studies showing the expression of ELL3 was associated with exosomal and non-exosomal microRNAs (Feng et al., 2019). Chen et al. (2020) demonstrated that PTPN22 was associated with the T stage and pathological grade of STAD. Zhang et al. (2003) delved into the roles of TPA, also known as PLAT, in the apoptosis of gastric cancer cells. They found that TPA inhibits PKB activity and causes its degradation, leading to apoptosis in gastric cancer cells.

Furthermore, it has been verified that NOX4 (Tang et al., 2018) and TGFB2 (Zhang and Li, 2016) exhibit elevated expression levels in tumor tissues relative to normal tissues. Furthermore, we obtained immunohistochemistry (IHC) staining images from GC and healthy stomach tissue associated with remaining RCD genes from the HPA database. The results indicated that there were variations in the protein expression levels of the remaining RCD genes between GC and healthy stomach tissues (Supplementary Figure S3; Supplementary Table S5).

The tumor immune microenvironment is a critical factor in predicting the responsiveness of GC to immunotherapy and evaluating its prognosis. The study conducted a comprehensive examination of immune infiltration and discovered that GC patients in the high-RCDI score cohort displayed a notable abundance of immune cells, such as NK cells, macrophages, and dendritic cells. Moreover, the association between immunotherapy indicators and immunotherapy datasets, along with the RCDI signature, suggests that persons with lower RCDI scores in GC are likely to have better immunotherapy outcomes. Our research findings suggest that RCDI has the potential to be a valuable biomarker for predicting genomic patterns and evaluating the efficacy of immunotherapy in patients with GC.

A significant limitation of this study is the lack of *in vitro* or *in vivo* experiments to directly confirm our results. Although bioinformatics analysis and computational methodologies are widely used, experimental validation remains an essential aspect of scientific research. By performing experiments, we can get valuable insights into the functional implications of observed patterns and improve the trustworthiness of our findings. Therefore, future research must give priority to conducting targeted experiments to verify and build upon the results of RCDI.

## 5 Conclusion

In general, our findings suggest that the RCDI signature can serve as a valuable tool for guiding treatment decisions and improving patient outcomes.

## Data Availability

The datasets presented in this study can be found in online repositories. The names of the repository/repositories and accession number(s) can be found in the article/Supplementary Material.
