# Supplementary material for: Unraveling the regulatory cell death pathways in gastric cancer: a multi-omics study
Source: Front Pharmacol. 2024 Sep 9;15:1447970. doi: 10.3389/fphar.2024.1447970 (PMC11417042; doi:10.3389/fphar.2024.1447970)
Supplement: Supplementary file 2 [file DataSheet1.docx]

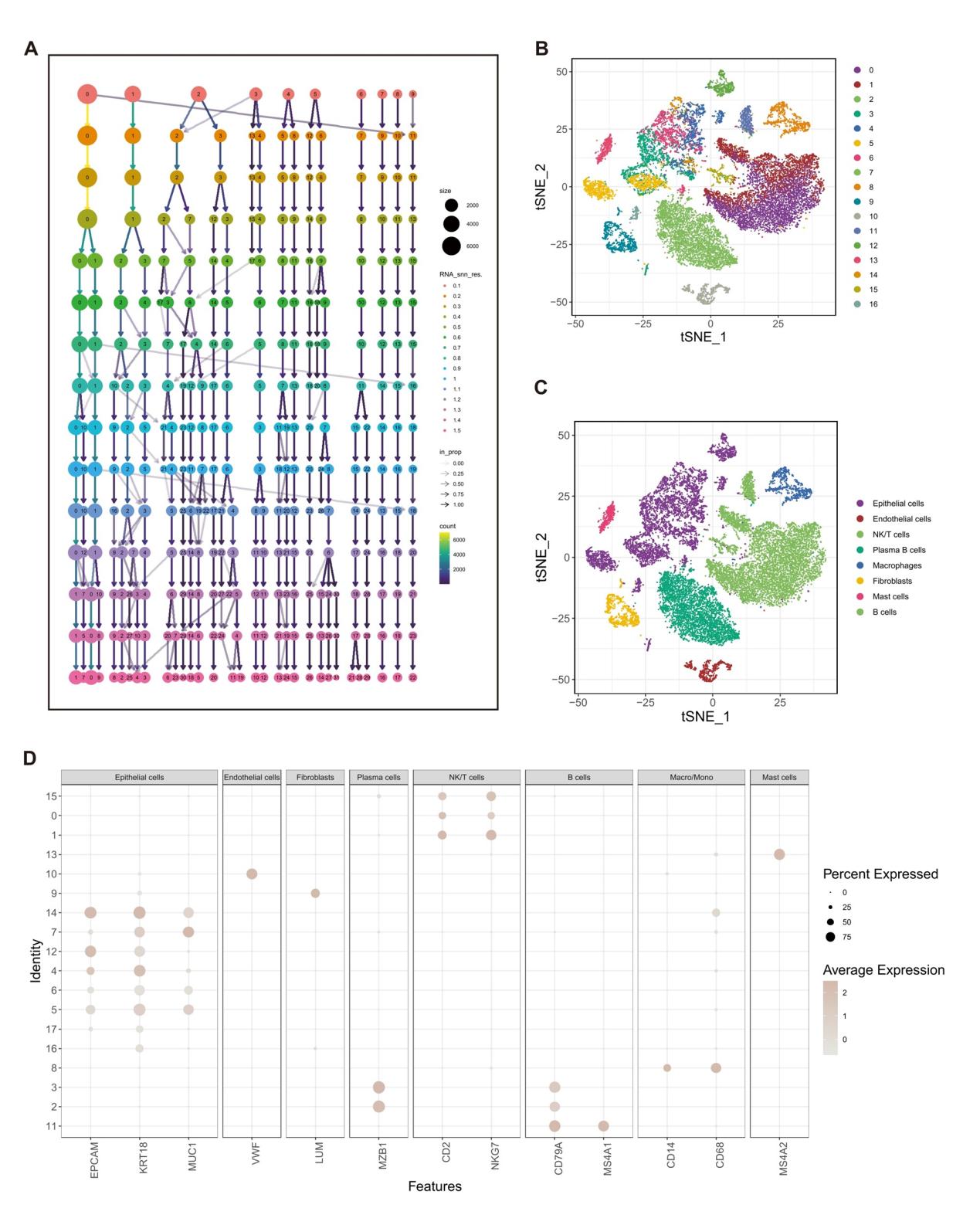


**SFigure 1 Annotation of scRNA-seq mate data**

1. The clustering tree displaying total scRNA-seq mate data analyzed at different resolutions. **(B)** The t-SNE plot displaying the cell clusters in the microenvironment of GC. **(C)** The t-SNE plot displaying the composition of cells in the microenvironment of GC. **(D)** Dot plot displaying expression of cell-type markers across cell clusters. Dot size indicates the percentage of expressed cells, colored by their relative expression levels


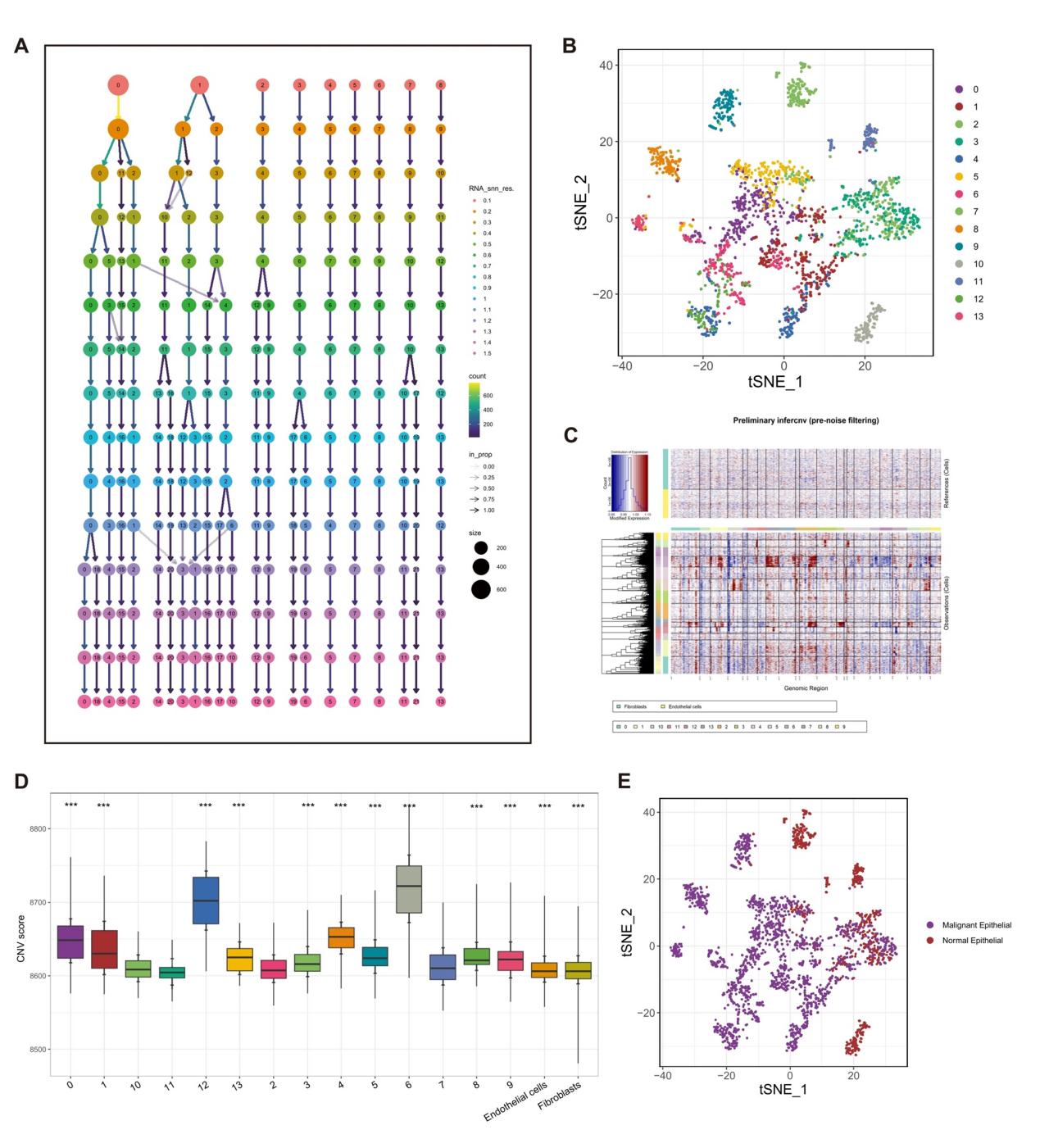


**SFigure 2 Distinguishing between malignant and normal cells in epithelial cells**

1. The clustering tree displaying total scRNA-seq mate data analyzed at different resolutions. **(B)** The t-SNE plot displaying the epithelial cell clusters in the microenvironment of GC. **(C)** Infercnv plotidentified malignant cells in epithelial cells.**(D)**The boxplot displaying the levels of CNV scores for each cluster of epithelial cells and the reference cells.**(E)** The t-SNE plot displaying the composition of cells in the microenvironment of GC.


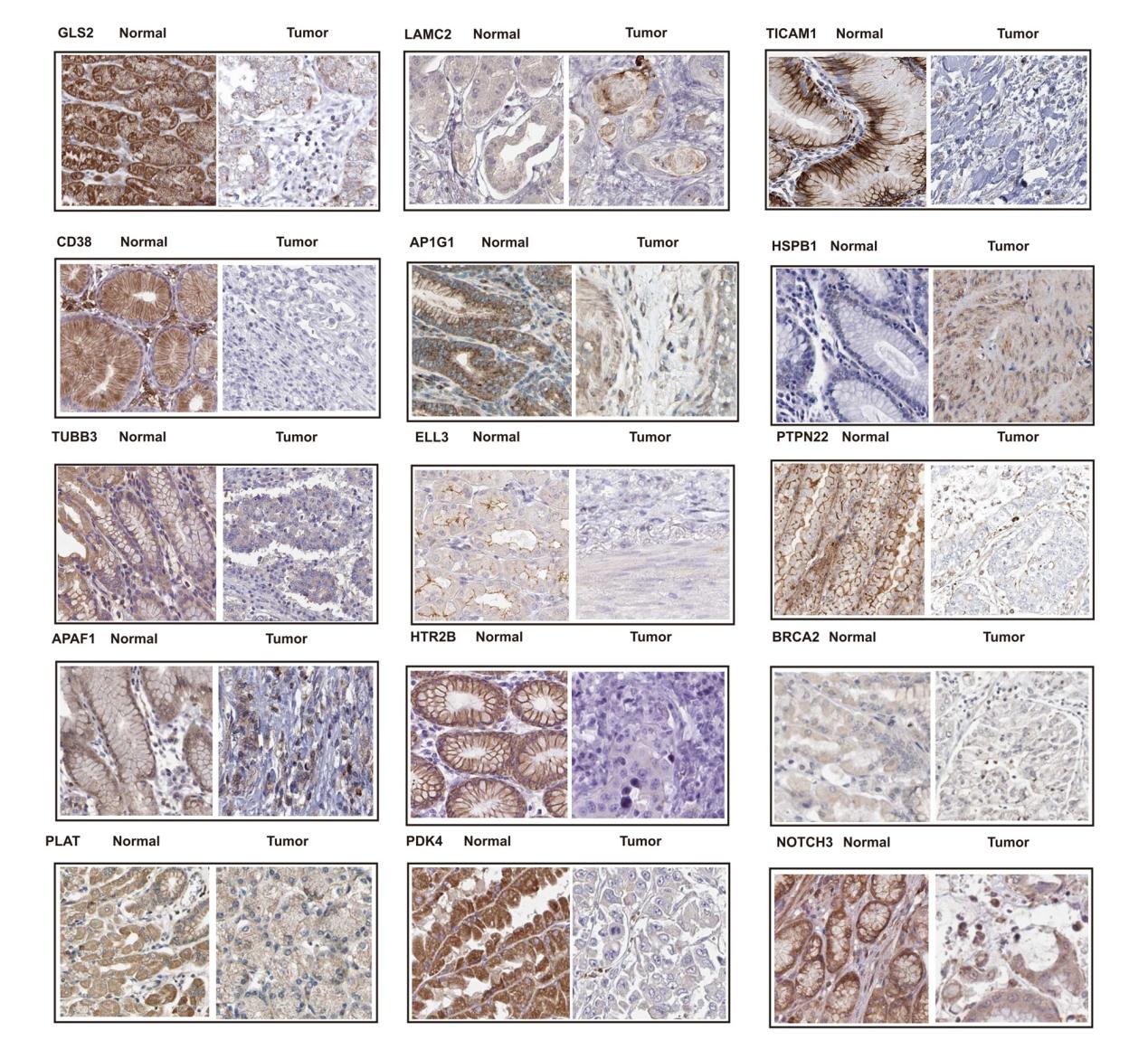


**SFigure 3 The immunohistochemistry (IHC) staining images of RCDI genes from GC and healthy stomach tissue in the HPA database.**
